# E2F4 regulatory program predicts patient survival prognosis in breast cancer

**DOI:** 10.1186/s13058-014-0486-7

**Published:** 2014-12-02

**Authors:** Sari S Khaleel, Erik H Andrews, Matthew Ung, James DiRenzo, Chao Cheng

**Affiliations:** 1 Department of Genetics, Geisel School of Medicine at Dartmouth, 1 Rope Ferry Road, Hanover, 03755 NH USA; 2 Department of Pharmacology & Toxicology, Geisel School of Medicine at Dartmouth, 1 Rope Ferry Road, Hanover, 03755 NH USA; 3 Institute for Quantitative Biomedical Sciences, Geisel School of Medicine at Dartmouth, One Medical Center Drive, Lebanon, 03766 NH USA; 4 Norris Cotton Cancer Center, Geisel School of Medicine at Dartmouth, One Medical Center Drive, Lebanon, 03766 NH USA

## Abstract

**Introduction:**

Genetic and molecular signatures have been incorporated into cancer prognosis prediction and treatment decisions with good success over the past decade. Clinically, these signatures are usually used in early-stage cancers to evaluate whether they require adjuvant therapy following surgical resection. A molecular signature that is prognostic across more clinical contexts would be a useful addition to current signatures.

**Methods:**

We defined a signature for the ubiquitous tissue factor, E2F4, based on its shared target genes in multiple tissues. These target genes were identified by chromatin immunoprecipitation sequencing (ChIP-seq) experiments using a probabilistic method. We then computationally calculated the regulatory activity score (RAS) of E2F4 in cancer tissues, and examined how E2F4 RAS correlates with patient survival.

**Results:**

Genes in our E2F4 signature were 21-fold more likely to be correlated with breast cancer patient survival time compared to randomly selected genes. Using eight independent breast cancer datasets containing over 1,900 unique samples, we stratified patients into low and high E2F4 RAS groups. E2F4 activity stratification was highly predictive of patient outcome, and our results remained robust even when controlling for many factors including patient age, tumor size, grade, estrogen receptor (ER) status, lymph node (LN) status, whether the patient received adjuvant therapy, and the patient’s other prognostic indices such as Adjuvant! and the Nottingham Prognostic Index scores. Furthermore, the fractions of samples with positive E2F4 RAS vary in different intrinsic breast cancer subtypes, consistent with the different survival profiles of these subtypes.

**Conclusions:**

We defined a prognostic signature, the E2F4 regulatory activity score, and showed it to be significantly predictive of patient outcome in breast cancer regardless of treatment status and the states of many other clinicopathological variables. It can be used in conjunction with other breast cancer classification methods such as Oncotype DX to improve clinical outcome prediction.

**Electronic supplementary material:**

The online version of this article (doi:10.1186/s13058-014-0486-7) contains supplementary material, which is available to authorized users.

## Introduction

Cancer prognosis and treatment plans rely on a collection of clinicopathological variables that stratify cancers outcomes by stage, grade, responsiveness to adjuvant therapy, and so on. Despite stratification, cancer’s enormous heterogeneity has made precise outcome prediction elusive and the selection of the optimal treatment for each patient a difficult and uncertain choice. Over the past two decades, advances in molecular biology have allowed molecular signatures to become increasingly obtainable [1] and incorporated into determining cancer prognosis and treatment [2]. For some cancer types, like breast cancer, gene expression signatures are now routinely used prognostically, with many research groups having identified signatures that predict cancer outcome or consider if patients will benefit from adjuvant therapy following surgical resection [3]-[9]. Surprisingly, however, there is little overlap in genes between the various signatures within different tissues or the same tissue (for example, breast cancer) raising questions about their biological meaning. Furthermore, even with gene expression signatures’ successes in cancer outcome prediction, improvement is possible, as the majority of these signatures are applicable only to early-stage cancers without lymph node (LN) metastasis or even previous chemotherapy. As cancer is fundamentally a disease of genetic dysregulation, specifically analyzing a tumor’s regulatory actors, such as transcription factors (TFs), may provide additional prognostic insight [10],[11], since transcription factors are relatively universal among different cell lines when compared to the tissue-specific gene clusters from which most gene signatures are made.

TFs are proteins that relay cellular signals to their target genes by binding to the DNA regulatory sequences of these genes and modulating their transcription [12]. They play major roles in many diverse cellular processes [13]-[17]. Unsurprisingly, aberrant expression or mutation of TFs or of their upstream signaling proteins has been implicated in an array of human diseases, including cancer [18]-[20]. Given their central regulatory functions, monitoring of TFs is widely regarded as a potentially useful and biologically sensible method for the prediction of cancer and disease outcome [1].

While differences in the transcriptional expression level of a TF do not necessarily correspond to differences in its regulatory activity, differences in the expression levels of a TF’s target genes do [21]-[23]. We have previously developed an algorithm to make this inference of a TF’s regulatory activity from the expression of its target genes, called REACTIN (REgulatory ACTivity INference) [24]. REACTIN can calculate the activity level of a TF on each individual sample in a given dataset. By calculating these levels and generating individual regulatory activity scores (iRASs) for a given TF and sample, REACTIN reveals a given TF’s activity level for each individual sample relative to all others in a dataset, thereby enabling the incorporation of a TF’s activity level into regression-based analyses. For example, by combining these iRAS TF activity levels with survival data, Cox proportional hazard (PH) models can be employed to examine how TF activity levels correlate with survival outcomes.

In this study, we define an E2F4 signature based on its target genes identified by chromatin immunoprecipitation sequencing (ChIP-seq) experiments. Based on the signature, E2F4 activity is inferred in breast cancer samples and used for predicting clinical outcomes. We focus on the E2F4 signature, because we have previously identified it as being prognostic in breast cancer in a large-scale computational screening analysis [24]. Further, in other work we have found E2F4’s activity level to be the most important of all TFs in predicting cell cycle phase in the HeLa and K562 cell lines, suggesting an essential role for E2F4 in cell cycle regulation [25]. Beyond our work, it is broadly considered that E2F4 plays an important role in both cell cycle arrest [26] and in modulating cell proliferation [27]. Furthermore, transgenic mice overexpressing E2F4 develop tumors, and mutated E2F4 has been reported in several types of cancers, including cancers of the gastrointestinal tract and prostate [26],[28], suggesting a broad tumorigenic role for E2F4.

Using E2F4 with our REACTIN method and clinical outcome data, we examine E2F4’s regulatory activity in detail as a predictor of survival outcome for breast cancer. With a collection of eight publicly available datasets containing gene expression and survival data for over 1,900 breast tumor samples, we show that E2F4 regulatory activity is strongly prognostic and remains so even after adjusting for other molecular markers, clinicopathological variables, clinical risk scores, Oncotype DX stratification, and differences in patient treatment. E2F4 activity level also correlates with classification assignment of breast cancers into their intrinsic subtypes. Extending beyond breast cancer, we preliminarily analyze E2F4 regulatory activity levels in bladder, colon, non-small cell lung, glioblastoma, acute myeloid leukemia, and Burkitt’s lymphoma cancer types, respectively, and find that they appear prognostic in colon, glioblastoma, and bladder cancer. E2F4 regulatory activity level predicts breast cancer survival outcome and may be of use in augmenting prognosis in cancer types.

## Methods

### Collection of gene expression cell cycle data

Human cell cycle gene expression profiles collected in HeLa S3 cells using two-channel cDNA arrays [29] were downloaded from the National Center for Biotechnology Information (NCBI) Gene Expression Omnibus (GEO, [30]; GSE3497). The dataset contained expression profiles from five independent time courses, from which we used the course with the largest number of time points (N = 48) for our analysis.

### Collection of gene expression and patient clinical and survival data

Using the collated Ur-Rehman *et al*. [31] meta-analysis as a guide, the ROCK, GEO [30], and National Institutes of Health PubMed [32] databases were queried to access and download all publicly available breast cancer gene expression datasets for which standard clinical data (age at diagnosis, estrogen receptor (ER) status, tumor size, grade, and LN involvement) and survival outcome data were present for a minimum of 150 samples. Depending on the availability from the original publications, either distant metastasis-free survival (DMFS) or relapse-free survival (RFS) was used as survival outcome. This resulted in the collection of 1,902 unique breast cancer samples across eight different datasets and on both one- and two-channel arrays (Table 1).

**Table 1:** **List of cancer datasets used in this analysis**

GSE ID	Platform	Cancer type	Number of samples	Source
-	cDNA two channel	Breast	295	Vijver *et al*., 2002 [4]
GSE1456	HG-U133A	Breast	159	Pawitan *et al*., 2005 [8]
GSE2034	HG-U133A	Breast	286	Wang *et al*., 2005 [5]
GSE2990	HG-U133A	Breast	177	Sotiriou *et al*., 2006 [6]
GSE3494	HG-U133A	Breast	260	Miller *et al*., 2005 [7]
GSE6532	HG-U133A	Breast	327	Loi *et al*., 2008 [33]
GSE7390	HG-U133A	Breast	198	Desmedt *et al*., 2007 [34]
GSE11121	HG-U133A	Breast	200	Schmidt *et al*., 2008 [35]
GSE13507	Illumina beadchip	Bladder	256	Kim *et al*., 2010 [36]
GSE13041	HG-U133A	Glioblastoma	191	Lee *et al*., 2008 [37]
GSE8894	HG-U133 Plus 2	Non-small cell lung	138	Lee *et al*., 2008 [38]
GSE17536	HG-U133 Plus 2	Colon	177	Smith *et al*., 2010 [39]
GSE425	cDNA two channel	Acute myeloid leukemia	119	Bullinger *et al*., 2004 [40]
GSE4475	HG-U133A	Burkitt’s lymphoma	221	Hummel *et al*., 2006 [41]

For each sample, composite predictive measures derived from clinical data, the Nottingham Prognostic Index (NPI) [42] and Adjuvant!Online [43] scores, were calculated and recorded. The Adjuvant! risk score of ‘high’ or ‘low’ was derived from the Adjuvant!Online numerical scores following the procedure in [34], while the NPI risk scores of ‘low’, ‘medium’, or ‘high’ were derived from the standard numerical score ranges of <3.4 , 3.4 to 5.4, and >5.4, respectively [42]. The combined table with all sample metadata can be found in Table S1 in Additional file 1.

In addition to breast cancer data, we collected gene expression and survival data for six other cancer types, including bladder cancer, glioblastoma, non-small cell lung cancer (NSCLC), colon cancer, acute myeloid leukemia (AML) and Burkitt’s lymphoma (Table 1).

### Definition of the E2F4 target gene signature

All publicly available E2F4 ChIP-seq datasets were accessed and downloaded, resulting in the collection of E2F4 chromatin-binding data in the GM06900, HeLa, and K562 cell lines [27],[44]. With a threshold false discovery rate of 1%, the TIP probabilistic method [45] was used to determine the candidate target genes of E2F4 in each cell line, resulting in the identification of 428, 438, and 429 target genes in the GM06990, HeLa, K562 and cells lines, respectively. The 199 identified target genes shared across the three cell lines were selected as the E2F4 target gene signature.

### Calculation of iRASs for E2F4 in cancer samples

The REACTIN algorithm, as introduced and previously described in [24], was applied to all collected cancer samples using the E2F4 target gene signature and with a minimum of 10,000 permutations. Briefly, REACTIN sorts the relative expression levels of all genes in a given sample and generates two cumulative distribution functions to summarize the expression levels of a target gene set and non-target gene set of a chosen TF - here, E2F4. REACTIN then uses the differential scores, calculated by comparing the two functions, to obtain the iRAS for E2F4 in each tumor sample. These resulting iRASs are scores similar to the values of the D-statistic in the Kolmogorov-Simonov test (KS test) and reflect the regulatory activity of E2F4 in a sample, with a higher iRAS value indicating a higher E2F4 regulatory activity as compared to a lower iRAS value.

For gene expression data measured by two-channel arrays, the expression levels of genes are represented as relative values: the log ratios of genes in a sample with respect to a control. In this case, the expression data can be directly used as input to the REACTIN method. However, for gene expression data from one-channel arrays, the absolute expression levels of genes are provided, which cannot be directly taken as input. To manage this problem, we performed gene-wise median normalization to convert the data into relative expression values. Specifically, we calculated median expression level for each gene across all samples and subtracted this median from all values. This median normalization was performed in log-transformed absolute expression values, thus making post-normalization data somewhat similar to the log ratios captured by two-channel arrays.

### Survival analyses

Cox PH models were used to examine if E2F4 activity correlated with patient survival outcomes. Both univariate and multivariate regression models with E2F4 iRASs alone, or E2F4 iRASs plus confounding variables (ER status, tumor size, grade, patient age, and so on), respectively, were investigated. Where indicated, E2F4 iRASs were dichotomized into positive score and negative score groups, enabling E2F4 iRASs to be treated as a binary variable throughout the analyses. Kaplan-Meier survival curves derived from the Cox PH models were also generated. For the breast cancer samples, analyses were performed both within each individual dataset and across the aggregated dataset derived from all individual datasets pooled together, as indicated. Analyses were performed in R using the ‘survival’ package, specifically using the ‘survreg’ and ‘coxph’ functions to construct the Cox PH models and the ‘survdiff’ function to compare the difference between two survival curves.

### Determination of intrinsic subtypes of breast cancer samples

Breast cancer samples were classified into the five intrinsic subtypes - basal-like, luminal A, luminal B, HER2-enriched, and normal-like [46] - using the PAM50 algorithm [47] after having their gene expression values median-centered as recommended [48]. Namely, Spearman correlation coefficients between the median-centered expression values in each sample and the provided PAM50 centroids for each of the five intrinsic subtypes were calculated. Samples were assigned to the subtype for which they had the highest Spearman correlation coefficient. Samples with correlations less than 0.1 for all subtypes were excluded from subsequent analysis.

### Oncotype DX analysis

The recurrence scores of breast cancer samples (ER-positive, LN-negative) were calculated using a 21-gene signature proposed by Oncotype DX [49]. Based on the scores, samples are stratified into low-, intermediate- and high-risk groups. The R package ‘genefu’ was used to implement the Oncotype DX analysis.

## Results

### The E2F4 target gene signature contains cell cycle regulators and is enriched for genes that correlate with patient survival

**Figure 1:**
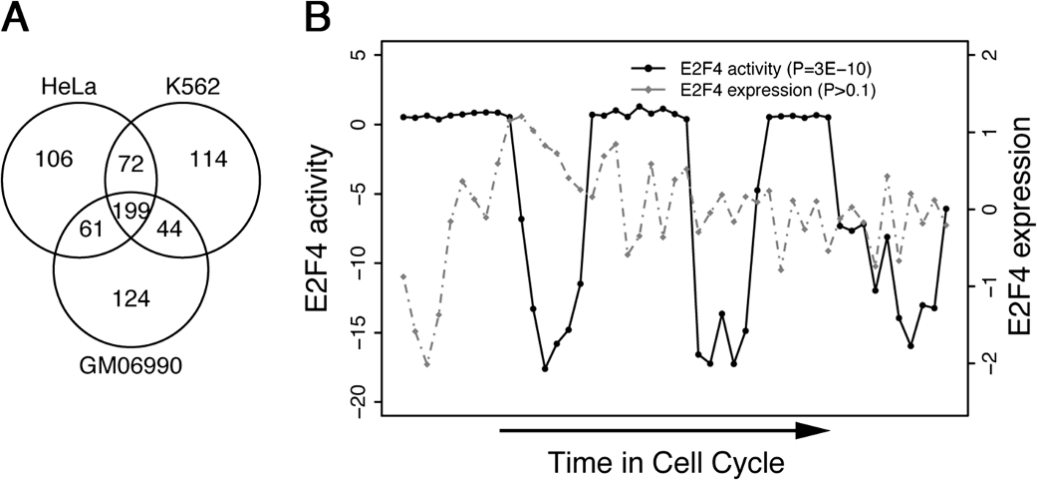
**E2F4’s target gene signature consists of 199 genes and reflects its activity in the cell cycle. (A)** A Venn diagram of target genes identified by ChIP-seq experiments in the HeLa, K562, and GM06900 cell lines. Using a *P* value <0.01 threshold, 199 genes are found to be targeted by E2F4 across all three cell lines. **(B)** E2F4 activity and expression levels throughout the cell cycle in HeLa S3 cells. Activity is calculated as the regulatory activity score (RAS) and expression is calculated in log ratio from cDNA array. The inferred E2F4 activity derived from RAS (solid black line), but not the E2F4 expression level (hashed gray line), is significantly periodic during the cell cycle. ChIP-seq, chromatin immunoprecipitation sequencing.

Leveraging E2F4 ChIP-seq data from experiments performed across HeLa and K562 [44] and GM06990 [27] cell lines, the TIP method [45] was used to identify E2F4 target genes in each cell line at a *P* value <0.01 confidence level. A total of 438, 429, and 428 target genes were identified in the HeLa, K562 and GM06990 cell lines, respectively, of which 199 were found to overlap across the three cell lines (Figure 1A). This shared group was defined as the E2F4 target gene signature. Examination of this gene signature using DAVID Functional Annotation Clustering [50] against a *Homo sapiens* gene background produced 58 clusters related to cell cycle regulation, mitosis, and microtubule organization; kinetochore; DNA repair; DNA replication; nucleoplasm; meiotic cell cycle, and nucleotide binding (Table S2 in Additional file 2). These cluster categories match the known important role played by E2F4 in cell cycle regulation, arrest, and/or progression [26],[27].

To preliminarily examine how these 199 E2F4 target genes might relate to survival, we next compared the correlation of their expression with survival to that of all genes in an initial dataset, van de Vijver *et al*. [4]. van de Vijver *et al*. measured the expression of a total of 10,333 genes, 102 of which are contained in the 199 E2F4 target gene signature. For all 10,333 genes, we carried out Cox regression analysis and found 751 of them to be significantly correlated with patient survival times (disease-free survival (DFS) time). Of these genes, 58 were among the 102 E2F4 targets, yielding an enrichment of 8-fold (751/10,333 vs. 58/102; *P* = 8e-40, Fisher’s exact test). After taking confounding factors such as ER status and positive LN involvement into account in the model, we identified 83 significant genes, 17 of which were E2F4 targets, yielding an enrichment of 21-fold (83/10,333 vs. 17/102; *P* = 2e-18, Fisher’s exact test). These results indicate that our E2F4 target gene signature is enriched for genes with predictive ability for patient survival in breast cancer.

### E2F4 iRASs outperform E2F4 expression levels as markers of cell cycle phase

To test the E2F4 target gene signature as an indicator of E2F4’s regulatory activity, we compared it to E2F4’s mRNA expression level in how it correlates to cell cycle phase in a HeLa S3 cell cycle dataset [29]. As E2F4 is a known critical cell cycle regulator, its activity cycles with cell cycle phase. Using REACTIN and E2F4’s target gene signature, we calculated the iRASs of E2F4 throughout the cell cycle. These iRASs show a significant periodical pattern (*P* = 3e-10, Fisher’s G test), while the expression levels of E2F4 do not (*P* >0.1, Fisher’s G test) (Figure 1B). We conclude that REACTIN-derived E2F4 iRASs more accurately reflect E2F4 regulatory activity than E2F4 expression levels do.

### E2F4 iRASs predict breast cancer survival prognosis

**Figure 2:**
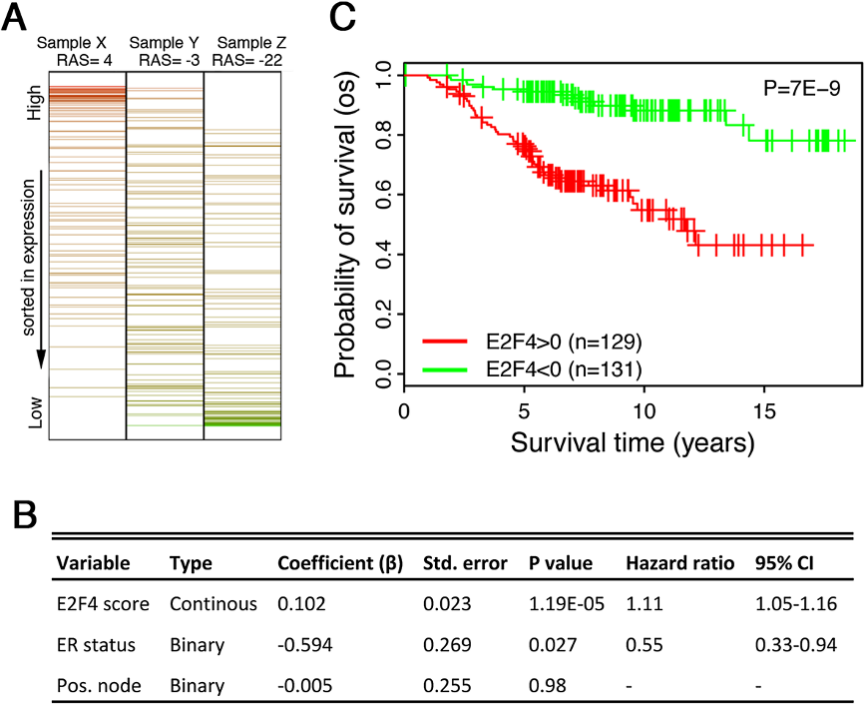
**E2F4 activity predicts patient survival in the van de Vijver breast cancer dataset. (A)** The E2F4 iRAS derives from the relative expression levels of E2F4’s target genes in individual samples. If its target genes are relatively highly expressed a larger iRAS results (Sample X), while lower average expression yields a lower iRAS (Sample Z). **(B)** In a Cox PH model, E2F4 iRAS significantly predicts patient survival even after adjusting for ER and LN lymph node status. **(C)** Patients with positive E2F4 scores (red curve) show significantly shorter survival times than those with negative E2F4 scores (green curve). Vertical hash marks indicate points of censored data. **(B)** and **(C)** derive from the van de Vijver dataset with overall survival (OS) as the endpoint. ER, estrogen receptor; iRAS, individual regulatory activity score; LN, lymph node; PH, proportional hazard.

We have previously shown that E2F4 activity inferred from expression of all genes predicts patient survival prognosis of breast cancer patients [24]. As a first test of our REACTIN method restricted to the E2F4 target gene signature, we paralleled this analysis here (Figure 2) using the same dataset [4]. For each breast cancer sample, an E2F4 iRAS was generated using REACTIN based on the sorted relative expression levels of the E2F4 target genes in the sample (Figure 2A). We compared the survival prediction with these iRASs scores to survival prediction with two commonly considered pathological variables: LN status (positive or negative) and ER status. Looking at patient outcome data, a Cox PH model shows that E2F4 iRASs improve survival prediction over ER and LN status alone (Figure 2B; *P* = 1e-5). After dichotomizing E2F4 iRASs into two groups of high activity, E2F4 iRAS >0 and low activity, E2F4 iRAS <0, a Kaplan-Meier plot comparing the two groups recapitulates this finding (Figure 2C; significance of difference between curves, *P* = 7e-9), with the E2F4 iRAS >0 group associated with worse prognosis. In contrast, the expression level of E2F4 itself does not significantly predict survival prognosis (*P* >0.4, data not shown), mirroring the Figure 1 finding that activity scores are a better indicator of E2F4 function than expression levels alone.

**Figure 3:**
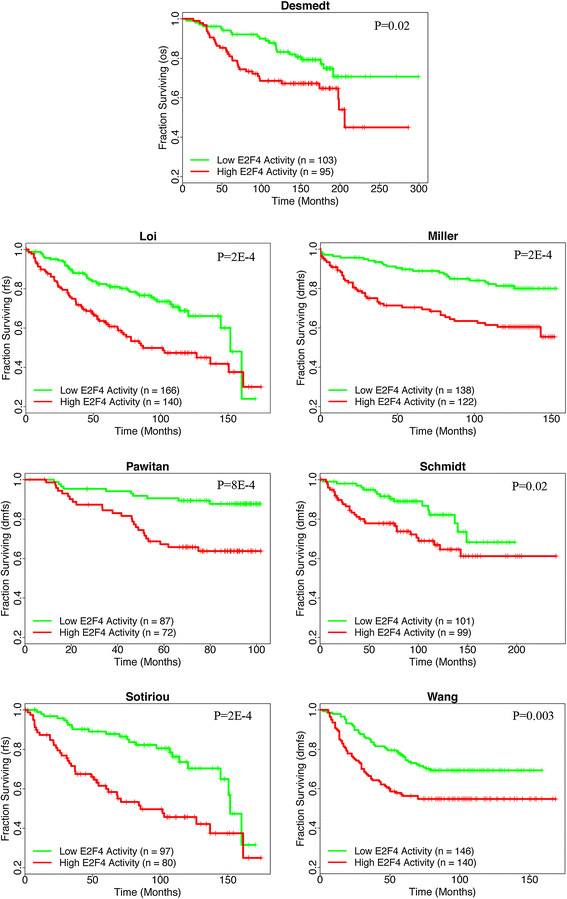
**E2F4 activity predicts patient survival prognosis in an additional seven independent breast cancer datasets.** Patients with positive E2F4 scores (red curve) show shorter survival times than those with negative E2F4 scores (green curve) across all datasets (all *P* values <0.05, log-rank test). Vertical hash marks indicate points of censored data. Survival endpoints are as indicated on the vertical axes: overall survival (OS); relapse-free survival (RFS); distant metastasis-free survival (DMFS).

To ensure that these results were not limited to the van de Vijver dataset, we obtained all additional publicly available breast cancer datasets for which survival and clinicopathological data were available for at least 150 samples (Table 1). As with the samples in the van de Vivjer dataset, we calculated iRASs for each sample and dichotomized them into high E2F4 activity (E2F4 iRAS >0) and low E2F4 activity (E2F4 iRAS <0) groups. Kaplan-Meier survival plots were then generated separately for each dataset, using as the survival endpoint whichever variable (overall survival, relapse-free survival, or distant metastasis-free survival) was most complete. In all seven of the datasets, E2F4 iRASs significantly predict survival outcome (all *P* values <0.05, Figure 3). As with the van de Vijver dataset, higher E2F4 activity (red curves) is predictive of worse survival prognosis.

Moreover, we carried out a similar analysis in the breast cancer metadata downloaded from the ROCK database, which provides normalized gene expression profiles and clinical information for 1,570 breast cancer samples. We calculated the E2F4 iRASs for all samples and dichotomized them into positive and negative groups. Survival analysis indicates that the RFS times of the positive groups are significantly shorter than those of the negative groups (*P* = 4e-8). After controlling for many clinical variables including patient age, tumor size, grade, ER status and LN status, the E2F4 iRAS is still highly significant in predicting patient RFS times (*P* = 6e-6) in a Cox survival regression model.

### E2F4 iRASs remain predictive of survival prognosis after pooling and adjustment for clinicopathological data

**Figure 4:**
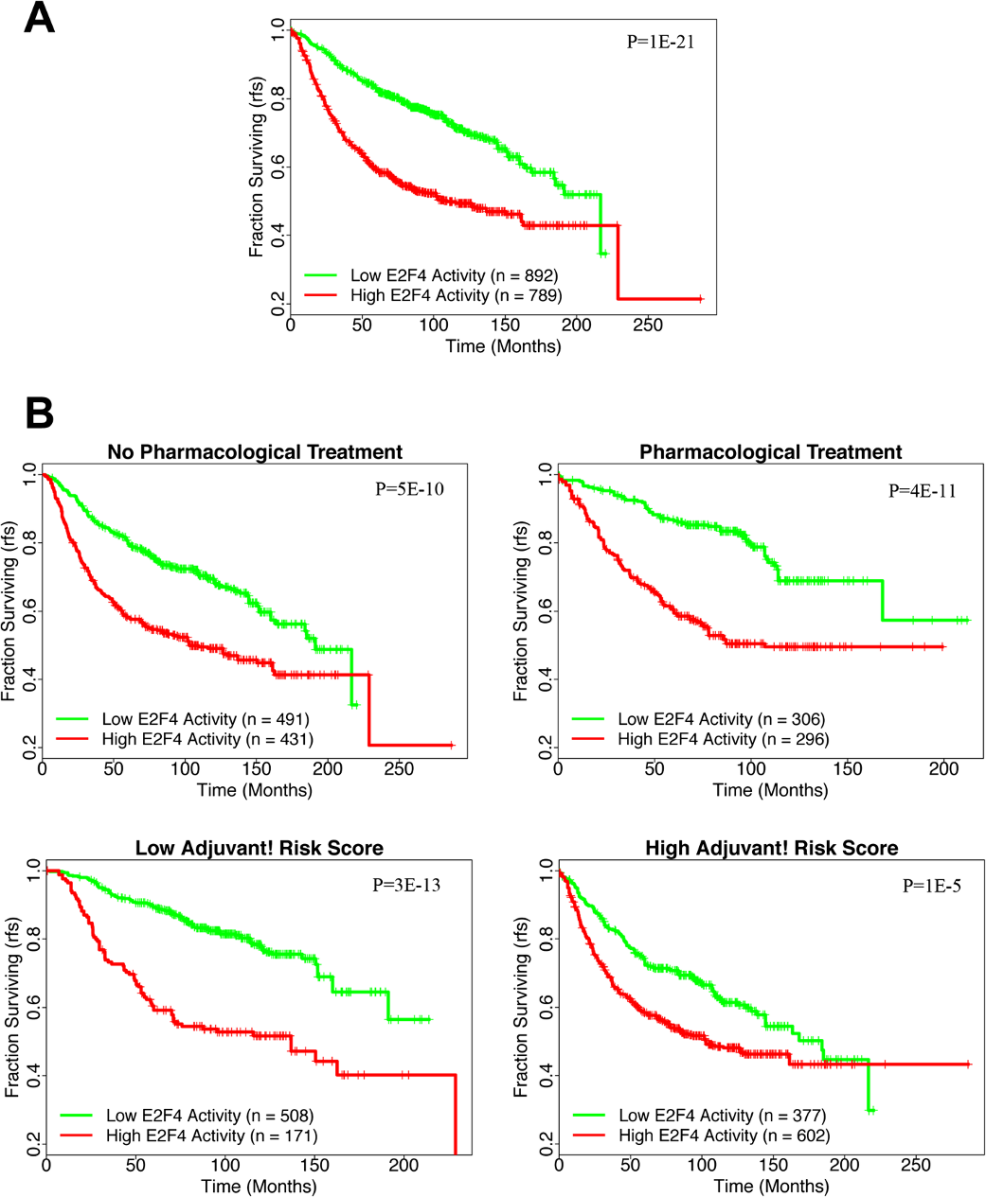
**Kaplan-Meier plots of the pooled breast cancer datasets, both unstratified (A) and stratified (B). (A)** As with the unpooled results, positive E2F4 scores (red curve) show shorter survival times than those with negative E2F4 scores (green curve) across all datasets (*P* value = 1.43e-21, log-rank test). **(B)** After stratification by pharmacological treatment (top two plots) or the Adjuvant! composite clinical risk score (bottom two plots), E2F4 iRASs remain predictive of survival prognosis within each stratum (all *P* values <0.001). Vertical hash marks indicate points of censored data. Survival endpoint is relapse-free survival (RFS) as indicated on the vertical axes. iRASs, individual regulatory activity scores.

Based on our results with individual breast cancer datasets, we decided to test REACTIN on a larger dataset, as the increased sample size from pooling would enable stratification and adjustment for other variables. Since iRASs are normalized values, they may be pooled to conduct aggregate analyses across data points. Combining together the samples from all eight breast cancer datasets, a Kaplan-Meier plot of the pooled data recapitulates the previous Figures 2 and 3 findings (Figure 4A, significance of difference between curves, *P* = 1e-21). As detailed in the Methods section, clinical data (age at diagnosis, ER status, tumor size, tumor grade, and LN involvement) were collected for all breast cancer samples and used to calculate clinical risk scores using the NPI and Adjuvant! Online formulae. The pharmacological treatment status of each sample - whether chemotherapy and/or hormone therapy was used - was additionally recorded.

Inclusion of these clinicopathological covariates in Cox PH models of the pooled samples results in adjusted E2F4 iRAS hazard ratios (HRs) that are positive and statistically significant (Table 2). Regardless of model chosen (Table 2A, B, and C), E2F4 iRASs significantly predict survival outcome, with a high E2F4 iRAS resulting in a worse survival prognosis than low E2F4 iRAS data points (HRs >1.00, *P* values <0.001 in all cases). Graphically, Kaplan-Meier plots of the pooled data, stratified by pharmacological treatment status and composite clinical risk, exhibit these findings as well (Figure 4B). E2F4 iRASs provide additional prognosis prediction beyond the commonly collected clinicopathological variables alone.

**Table 2:** **Cox PH models of E2F4 iRAS hazard ratios (HRs) adjusted for clinicopathological data**

**A**					
Variable	Type	Hazard ratio	Std. error	95% CI	***Pvalue***
E2F4 iRAS (high vs. low)	Binary	2.013	0.108	1.63 - 2.49	8.54E-11
Age	Continuous	1.002	0.004	0.99 - 1.01	0.6890
ER status (+ vs. -)	Binary	1.061	0.113	0.85 - 1.33	0.6029
Grade	Ordinal	1.157	0.074	1.01 - 1.34	0.0475
Size	Continuous	1.013	0.004	1.01 - 1.02	0.0001
Lymph node status (+ vs. -)	Binary	1.407	0.149	1.05 - 1.88	0.0215
Pharmacological treatment	Binary	0.651	0.148	0.49 - 0.87	0.0037
**B**					
**Variable**	**Type**	**Hazard ratio**	**Std. error**	**95% CI**	***P*** **value**
E2F4 iRAS (high vs. low)	Binary	1.9013	0.0918	1.59 - 2.28	2.57E-12
Adjuvant! risk score (low vs. high)	Binary	0.6799	0.1001	0.56 - 0.83	0.0001
Pharmacological treatment	Binary	0.8362	0.091	0.70 - 0.99	0.0493
**C**					
**Variable**	**Type**	**Hazard ratio**	**Std. error**	**95% CI**	***P*** **value**
E2F4 iRAS (high vs. low)	Binary	1.86177	0.10	1.52 - 2.27	1.12E-09
NPI score	Continuous	1.29314	0.05844	1.15 - 1.45	1.09E-05
Pharmacological treatment	Binary	0.76527	0.09927	0.63 - 0.93	0.0070

### E2F4 iRASs predict patient survival prognosis in ER-positive, PR-positive, and MYC-negative histological subtypes

**Figure 5:**
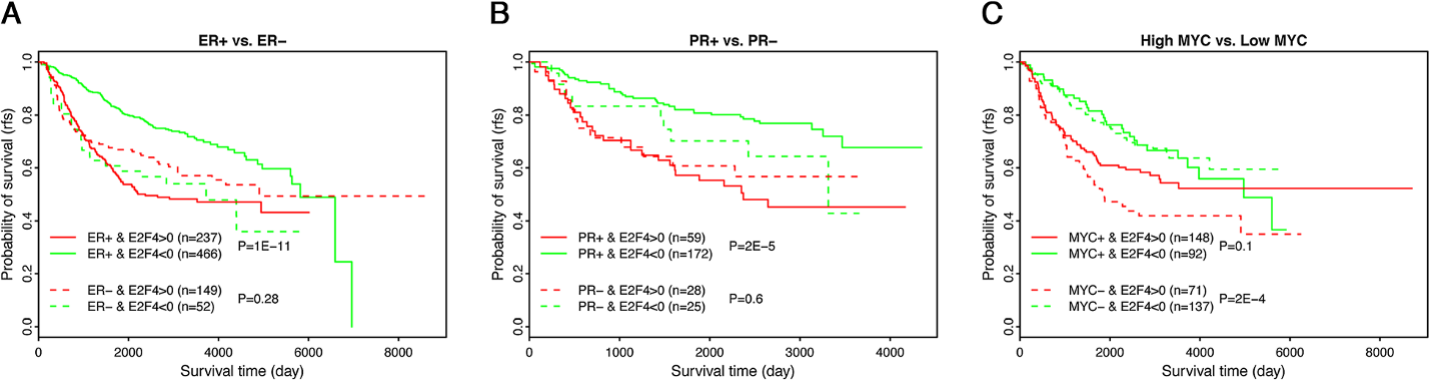
**Application of the E2F4 signature for predicting patient survival times in different histological subtypes.** Note that E2F4 signature is effective in ER+ but not in ER- samples **(A)**, in PR+ but not in PR- samples **(B)**, and in low MYC but not in high MYC samples **(C)**. ER, estrogen receptor; PR, progesterone receptor.

A tumor’s histological subtype is a key factor in planning breast cancer therapy. Stratifying tumors by ER subtype, we found that E2F4 regulatory activity was significantly correlated with survival in patients with ER-positive tumors (*P* = 6e-12), but not in ER-negative ones (*P* > 0.1) (Figure 5A). Furthermore, an examination of E2F4 activity distribution in ER-positive versus ER-negative patients showed significantly lower levels of E2F4 activity in the ER-negative group (*P* = 3e-10, Wilcoxon rank-sum test) (Figure S1 in Additional file 3). A similar pattern was seen with progesterone receptor (PR) status, where E2F4 was significantly correlated with survival in PR-positive (*P* = 2e-5) but not PR-negative patients (*P* >0.1) (Figure 5B). Stratification by MYC expression found E2F4 iRASs to be significantly correlated in MYC-negative (*P* = 2e-4) but not MYC-positive (*P* = 0.1) patients (Figure 5C).

### E2F4 iRASs correlate with the survival prognosis of intrinsic breast cancer subtypes

**Figure 6:**
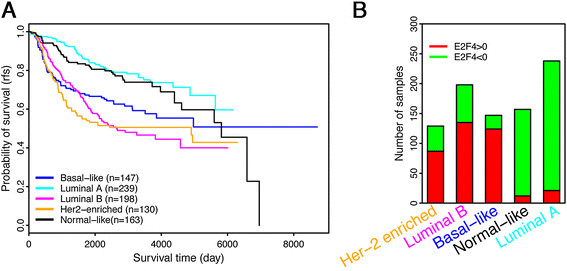
**E2F4 scores in different molecular subtypes of breast cancer. (A)** The survival curves of patients in five molecular subtypes. **(B)** The number of samples with positive (red) and negative (green) E2F4 iRASs in each molecular subtype. For the subtypes with relatively good prognosis, luminal A and normal-like, there is a high fraction of positive E2F4 iRAS samples, while the subtypes with relatively poor prognosis, HER2-enriched, luminal B and basal-like, have predominantly high fractions of negative E2F4 iRAS samples (A) compared to (B). iRASs, individual regulatory activity scores.

It has become increasingly understood that breast cancers segregate by gene expression into different intrinsic subtypes, with the assumption that cancers falling within the same subtype share a similar prognosis and suggested therapy method [51]. Several breast cancer subtypes have been identified, including luminal A, luminal B, HER2-enriched, basal-like, and normal-like cancers [47]. In a pooled analysis of the eight breast cancer datasets, a Kaplan-Meier plot of each sample classified into one of these intrinsic subtypes shows that subtypes have different survival prognoses (Figure 6A). Consistent with previous reports [47], the subtypes fall from good to poor prognosis in the order of luminal A, normal-like, basal-like, luminal B and HER2-enriched. Furthermore, the prognosis of these different molecular subtypes is strongly correlated with E2F4 iRAS: a high fraction of samples with positive E2F4 iRASs fall into the poor prognostic subtypes (HER2-enriched, luminal B and basal-like), whereas in good prognostic subtypes (luminal A and normal-like), the fraction of samples with a positive E2F4 iRAS is much lower (Figure 6B). These results indicate that the survival prognoses of different intrinsic subtypes can be at least partially reflected by the E2F4 regulatory program.

### Application of E2F4 signature to other cancers

**Figure 7:**
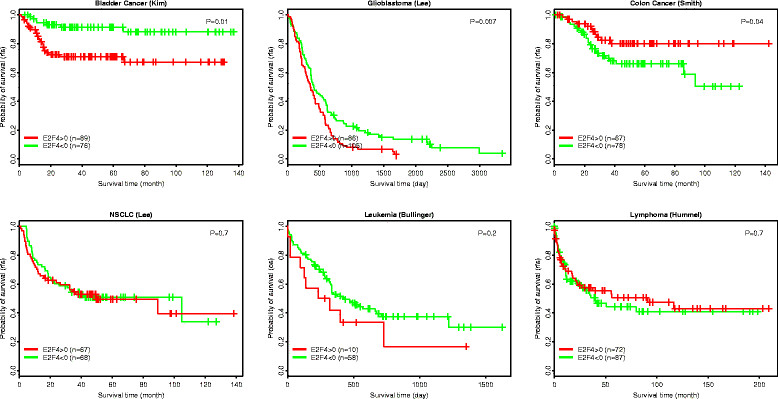
**Application of the E2F4 signature for predicting patient survival times in six cancer types.** In each cancer dataset, patients are stratified into a group with positive E2F4 activity and a group with negative E2F4 activity. The difference in their survival curves is calculated using the log-rank test.

The predictive power of E2F4 iRASs for breast cancer survival and the correlation of this signature with cell cycle phase encouraged us to test the effectiveness of the E2F4 signature for patient survival prediction in other cancers. E2F4 iRASs were calculated for samples in bladder cancer [36], colon cancer [39], NSCLC [38], glioblastoma [37], AML [40], and Burkitt’s lymphoma [41] datasets and used in conjunction with the available survival data to generate Kaplan-Meier plots (Figure 7). E2F4 activity level significantly correlates with survival time in bladder cancer (*P* = 0.01) and glioblastoma (*P* = 0.007), but not in NSCLC, AML or Burkitt’s lymphoma (*P* >0.05). In colon cancer, although the survival curves for E2F4 iRAS >0 group and iRAS <0 groups are fairly separated, the statistical difference is only moderately significant (*P* = 0.04). In addition, the survival times of the two groups are opposite to what we observed in other cancer types. More detailed analyses - in datasets with larger number of samples and with controlling for other factors - should be carried out before we can conclude the significance of the E2F4 signature in non-breast cancer types.

## Discussion

Several breast and other cancer prognostic methods rely on gene expression signatures as predictors of survival or the need for adjuvant therapy for a patient. While these methods have seen prognostic success and improved decision-making regarding patient treatment plans, they intrinsically suffer from problems of overfitting and multiple comparisons that raise questions of whether the genes selected in the signatures are of biological and etiological significance. Indeed, much concern has been raised about the small degree of overlap between different prognostic gene signatures and the degree to which the given microarray platform (Affymetrix vs. Agilent, and so on) affects the gene composition of each signature [52],[53].

In this manuscript, we present the result of an alternative and more robust method of deriving a gene prognostic signature: using ChIP-seq data from multiple cell lines, we identify a TF’s set of gene targets, whose differential expression in patient samples can be used to calculate the TF’s regulatory activity in these samples. By examining TF activity, biological significance of the signature is preserved. Further, by inferring this TF activity through the expression levels of its set of gene targets, the TF’s actual functional activity is assessed - TFs work via altering their target genes’ expression levels - and in a way that allows for the use of widely available microarray data and regardless of platform type. Hence, the signature is fundamentally derived from mechanistic relationships of genetic regulation and is easily measured with current techniques.

With E2F4 ChIP-seq data in the three cell lines of GM06990, K562, and HeLa S3, we have identified a set of 199 genes as significantly targeted (*P* <0.01) by E2F4 across the three cell lines (Figure 1A). To confirm this gene set’s ability to infer E2F4’s regulatory activity level, we have used the gene set in conjunction with REACTIN to generate E2F4 iRASs in a cell cycle phase dataset [29], finding that E2F4 iRASs exhibits a periodic pattern and greatly outperforms E2F4 gene expression level in correlating with cell cycle phase (Figure 1B). As E2F4 is a known critical regulator of the cell cycle [25], this result suggests that E2F4 iRASs reflect E2F4 functional activity and with much better accuracy than E2F4 gene expression level alone.

Using this method of generating E2F4 iRASs for given samples, we turned to breast cancer based on our prior work [24] to examine E2F4’s inferred functional activity through iRASs and its ability to predict survival prognosis. In all publicly available, sufficiently sized datasets containing survival data, we have found that E2F4 iRASs are significant predictors of survival outcome (Figures 2, 3 and 4A), with a higher E2F4 iRAS predicting shorter survival. Importantly, pooled analyses further show that this predictive power remains robust even after adjusting for clinicopathological data through Cox PH multivariate regression (Table 2) and stratification, including by pharmacological treatment status (Figure 4). In contrast, most of the currently available prognostic gene signature tools apply only to early-stage, untreated cancers. Interestingly, in all Cox PH models (Table 2), the E2F4 iRAS has the largest HR and smallest *P* value of all covariates, suggesting it not only provides prognostic prediction beyond the other variables but that it is additionally the most important driver of survival outcome. The finding that it remains prognostic even with stratification by treatment status further indicates that current pharmacological therapy does not disrupt the E2F4 functional program, perhaps suggesting a potential future treatment target.

Beyond examination with clinicopathological data, we examined E2F4 iRASs and their relationship to histological and molecular subtypes. We evaluated confounding of our results by patient ER and PR status, based on literature linking E2F4 with progression of estrogen-dependent breast cancer cell lines. Interestingly, E2F4 was a significant predictor of survival only in ER-positive and PR-positive and not ER-negative or PR-negative patients (Figure 5A and B), suggesting that E2F4’s regulatory activity plays a role in steroid-dependent but not steroid-independent cancers. A connection between ER-mediated regulation of the cell cycle and E2F4 has been previously suggested: Carrol *et al*. proposed a mechanism for anti-estrogen drug effects on cell cycle arrest involving the phosphorylation of E2F4, which then induced cell cycle arrest in the MCF7 breast cancer cell line [54]. Dhillon *et al*. showed that MCF7-CycE, a breast cancer cell line overexpressing cyclin E (which, in turn, binds E2F4), was capable of overriding tamoxifen-mediated growth arrest in comparison to its wild-type MCF7 counterpart [55]. These results imply a connection between E2F4 activity and ER-mediated effects on the cell cycle, which agree with our observation of ER status as a confounder of E2F4 activity in breast cancer survival.

Additionally, we have compared the results from the E2F4 signature and those from the Oncotype DX method [49] (Figure S2 in Additional file 4). From the Ur-Rehman breast cancer metadata, we selected 557 samples that were ER-positive, LN-negative and had known RFS information. We calculated the ‘recurrence score’ of these samples using the Oncotype DX method, and then divided samples into low- (200 samples), intermediate- (124 samples) or high- (233 samples) risk groups. Survival analysis indicates that the three groups are significantly different in their RFS (*P* = 2e-12). The E2F4 signature achieved comparable results in the same sample set - the iRAS >0 group (195 samples) displayed significantly shorter RFS than the iRAS <0 (362 samples) group (*P* = 8e-11). There is a correlation between the Oncotype DX groups and the E2F4 groups. In the high, intermediate and low Oncotype DX groups, the fraction of samples with E2F4 iRAS >0 are 64%, 19% and 11%, respectively, consistent with their expected prognosis. More importantly, our results indicate that the E2F4 signature can further improve the Oncotype DX classification results. When the 124 intermediate Oncotype DX samples were further stratified into iRAS >0 and iRAS <0 subgroups based on their E2F4 scores, the positive subgroup showed significantly shorter RFS than the negative subgroup (*P* = 0.0004). This suggests that E2F4 signature can be used in conjunction with the Oncotype DX system to achieve better performance.

Beyond E2F4 regulatory activity, cell proliferation can be captured by other molecular features. For instance, the Ki-67 protein (encoded by the MKI67 gene) is strictly associated with cell proliferation and has been used as a cellular marker for proliferation [56]. The prognostic value of it has been demonstrated in multiple tumor types including breast cancer [57]-[59]. Compared to the E2F4 signature that is based on multiple genes, however, the single gene MKI67 marker is not stable and generally shows lower predictive accuracy. Specifically, when samples are stratified into two groups with high and low MKI67 expression respectively, the two groups show significant survival difference with *P* = 0.0001 (data not shown), which is less predictive then the E2F4 signature (*P* = 7e-9, Figure 2C).

An emerging method for breast cancer prognosis relies on classification of breast cancer into intrinsic subtypes based on their gene expression profiles, with the five subtypes of normal-like, basal-like, luminal A and B, and HER2-enriched most frequently used (Parker *et al*. 2009 [47]). Analysis of E2F4 levels in these subtypes showed a significant variation among them, with lower levels of E2F4 activity seen in a greater fraction of cancer samples classified into intrinsic subtypes with better prognosis (Figure 6). Still, E2F4 could serve to improve the prognostic power of the current molecular classifications by adding an additional factor of classification: negative (low) versus positive (high) E2F4 activity. For example, when luminal B samples were further stratified into two groups based on E2F4 iRAS, the 50% samples with lower E2F4 activity exhibit significantly longer RFS times than the remaining 50% samples with higher E2F4 activity (*P* = 0.02).

Since our derived E2F4 signature was selected with the intent of being relatively tissue-independent, consisting of genes that play a role in cell cycle progression across three cell lines, we decided to evaluate E2F4 in six other cancers: bladder cancer, colon cancer, lung cancer (NSCLC), brain cancer (glioblastoma), leukemia (AML) and Burkitt’s lymphoma. Our results show that E2F4 was significantly correlated with survival time in bladder cancer, glioblastoma, and colon cancer, but not in NSCLC, AML or Burkitt’s lymphoma. These are preliminary results, and more investigations are needed to more precisely understand the role of the E2F4 regulatory program in tumorigenesis and progression of cancer types beyond breast cancer.

This study has several limitations. First, the numbers of effective samples in different cancer datasets are very different, which influence the power of statistical analysis. This also restricts the ability to examine the E2F4 signature in certain breast cancer subtypes accounting for only a small fraction of samples. Second, the breast cancer datasets used in this study are diverse in terms of sample selection, platforms for gene expression measurement, genetic background of patients, and treatment to patients. As such, it is difficult to identify the confounding variables in each dataset and correct their effect in prognosis. Finally, the quality of survival information in each dataset can vary considerably depending on the length of follow-up and other factors. This will also impact the results of the prognostic predictions.

Going forward, we aim to extend the application of the E2F4 signature to several directions. First, to refine the signature, we will select a subset of core genes from the E2F4 signature while keeping a comparable predictive power. Second, it will be useful to examine the effectiveness of this signature in more specific breast cancer subtypes, for example in ER+ LN+ (LN-positive) and ER+ LN-. Third, it will also be interesting to test whether the E2F4 signature can predict sensitivity to a specific drug or treatment, for example the CDK inhibitors, which can repress the E2F4 regulatory program. Finally, it will be useful to more thoroughly examine its effectiveness in other cancer types.

## Conclusions

This study presents a novel method of determining a signature for cancer prognosis that relies on a TF’s activity inferred from the differential expression of its target genes. We evaluated our method using E2F4, a well-known cell cycle regulator with an unclear role in cancer progression, and tested its predictive power for patient survival in breast cancer. Our results show a significant difference in survival between patients with positive and negative E2F4 activity scores, corresponding to high and low levels of E2F4 target gene expression, respectively. Survival is favored for patients with negative E2F4 activity scores, suggesting that the upregulation of E2F4 activity is associated with worse breast cancer prognosis. Comparison of hazard ratios between E2F4 activity scores and common prognostic scales, such as Adjuvant! and Nottingham, shows E2F4 to have a higher hazard ratio, suggesting it is a stronger predictor of tumor prognosis than these two clinicopathological-derived prognostic indices. Examining E2F4 activity scores within stratified groups, such as Oncotype DX risk strata, breast cancer intrinsic subtypes, and pharmacological treatment status, shows that E2F4 activity scores convey additional prognostic information within these strata. E2F4 activity level robustly predicts breast cancer patient survival across a variety of clinical contexts.

## Additional files

## Electronic supplementary material

Additional file 1: Table S1.: Clinical information and iRAS scores for all breast cancer samples used in this analysis. (XLS 650 KB)

Additional file 2: Table S2.: Gene Ontology categories that are enriched in the 199 E2F4 signature genes. (XLS 46 KB)

Additional file 3: Figure S1.: The distribution of E2F4 scores in all (the left panel), ER+ (the middle panel) and ER- (the right panel) breast cancer samples. (PDF 6 KB)

Additional file 4: Figure S2.: Application of E2F4 signature to predicting prognosis of ER+ node-breast cancer. Top left: Oncotype DX divides samples into high-, intermediate- and low-relapse risk groups. Top right: E2F4 signature divides patient into two groups with significant survival difference. Bottom left: E2F4 can further stratify the Oncotype-classified intermediate group into high- and low-risk groups. Bottom right: Within the Oncotype-classified intermediate group, patients with high and low Oncotype DX scores do not show significant difference in their survival times. (PDF 21 KB)

Below are the links to the authors’ original submitted files for images.

Authors’ original file for figure 1

Authors’ original file for figure 2

Authors’ original file for figure 3

Authors’ original file for figure 4

Authors’ original file for figure 5

Authors’ original file for figure 6

Authors’ original file for figure 7

## Abbreviations

AML: acute myeloid leukemia
ChIP-seq: chromatin immunoprecipitation sequencing
DFS: disease-free survival
DMFS: distant metastasis-free survival
ER: estrogen receptor
GEO: Gene Expression Omnibus
HR: hazard ratio
iRAS: individual regulatory activity score
LN: lymph node
OS: overall survival
NPI: Nottingham Prognostic Index
NSCLC: non-small cell lung cancer
PH: proportional hazard
PR: progesterone receptor
REACTIN: regulatory activity inference
RFS: relapse-free survival
TF: transcription factor

## References

[1] Liotta L, Petricoin E (2000). Molecular profiling of human cancer. Nat Rev Genet.

[2] Ginsburg GS, Willard HF (2009). Genomic and personalized medicine: foundations and applications. Transl Res.

[3] Veer LJ V ’t, Dai H, van de Vijver MJ, He YD, Hart AA, Mao M, Peterse HL, van der Kooy K, Marton MJ, Witteveen AT, Schreiber GJ, Kerkhoven RM, Roberts C, Linsley PS, Bernards R, Friend SH (2002). Gene expression profiling predicts clinical outcome of breast cancer. Nature.

[4] van de Vijver MJ, He YD, van’t Veer LJ, Dai H, Hart AA, Voskuil DW, Schreiber GJ, Peterse JL, Roberts C, Marton MJ, Parrish M, Atsma D, Witteveen A, Glas A, Delahaye L, van der Velde T, Bartelink H, Rodenhuis S, Rutgers ET, Friend SH, Bernards R (2002). A gene-expression signature as a predictor of survival in breast cancer. N Engl J Med.

[5] Wang Y, Klijn JG, Zhang Y, Sieuwerts AM, Look MP, Yang F, Talantov D, Timmermans M, Meijer-van Gelder ME, Yu J, Jatkoe T, Berns EM, Atkins D, Foekens JA (2005). Gene-expression profiles to predict distant metastasis of lymph-node-negative primary breast cancer. Lancet.

[6] Sotiriou C, Wirapati P, Loi S, Harris A, Fox S, Smeds J, Nordgren H, Farmer P, Praz V, Haibe-Kains B, Desmedt C, Larsimont D, Cardoso F, Peterse H, Nuyten D, Buyse M, Van de Vijver MJ, Bergh J, Piccart M, Delorenzi M (2006). Gene expression profiling in breast cancer: understanding the molecular basis of histologic grade to improve prognosis. J Natl Cancer Inst.

[7] Miller LD, Smeds J, George J, Vega VB, Vergara L, Ploner A, Pawitan Y, Hall P, Klaar S, Liu ET, Bergh J (2005). An expression signature for p53 status in human breast cancer predicts mutation status, transcriptional effects, and patient survival. Proc Natl Acad Sci U S A.

[8] Pawitan Y, Bjohle J, Amler L, Borg AL, Egyhazi S, Hall P, Han X, Holmberg L, Huang F, Klaar S, Liu ET, Miller L, Nordgren H, Ploner A, Sandelin K, Shaw PM, Smeds J, Skoog L, Wedren S, Bergh J (2005). Gene expression profiling spares early breast cancer patients from adjuvant therapy: derived and validated in two population-based cohorts. Breast Cancer Res.

[9] Hornberger J, Alvarado MD, Rebecca C, Gutierrez HR, Yu TM, Gradishar WJ (2012). Clinical validity/utility, change in practice patterns, and economic implications of risk stratifiers to predict outcomes for early-stage breast cancer: a systematic review. J Natl Cancer Inst.

[10] Eckhoff K, Flurschutz R, Trillsch F, Mahner S, Janicke F, Milde-Langosch K (2013). The prognostic significance of Jun transcription factors in ovarian cancer. J Cancer Res Clin Oncol.

[11] Haq R, Fisher DE (2011). Biology and clinical relevance of the micropthalmia family of transcription factors in human cancer. J Clin Oncol.

[12] Mitchell PJ, Tjian R (1989). Transcriptional regulation in mammalian cells by sequence-specific DNA binding proteins. Science.

[13] Helin K (1998). Regulation of cell proliferation by the E2F transcription factors. Curr Opin Genet Dev.

[14] Barkett M, Gilmore TD (1999). Control of apoptosis by Rel/NF-kappaB transcription factors. Oncogene.

[15] Ogino H, Ochi H, Reza HM, Yasuda K (2012). Transcription factors involved in lens development from the preplacodal ectoderm. Dev Biol.

[16] Kako K, Ishida N (1998). The role of transcription factors in circadian gene expression. Neurosci Res.

[17] Sanchez-Tillo E, Liu Y, de Barrios O, Siles L, Fanlo L, Cuatrecasas M, Darling DS, Dean DC, Castells A, Postigo A (2012). EMT-activating transcription factors in cancer: beyond EMT and tumor invasiveness. Cell Mol Life Sci.

[18] Darnell JE (2002). Transcription factors as targets for cancer therapy. Nat Rev Cancer.

[19] Suva ML, Riggi N, Bernstein BE (2013). Epigenetic reprogramming in cancer. Science.

[20] Nebert DW (2002). Transcription factors and cancer: an overview. Toxicology.

[21] Cheng C, Yan X, Sun F, Li LM (2007). Inferring activity changes of transcription factors by binding association with sorted expression profiles. BMC Bioinformatics.

[22] Rhodes DR, Kalyana-Sundaram S, Mahavisno V, Barrette TR, Ghosh D, Chinnaiyan AM (2005). Mining for regulatory programs in the cancer transcriptome. Nat Genet.

[23] Cheng C, Li LM (2008). Systematic identification of cell cycle regulated transcription factors from microarray time series data. BMC Genomics.

[24] Zhu M, Liu CC, Cheng C (2013). REACTIN: regulatory activity inference of transcription factors underlying human diseases with application to breast cancer. BMC Genomics.

[25] Cheng C, Ung M, Grant GD, Whitfield ML (2013). Transcription factor binding profiles reveal cyclic expression of human protein-coding genes and non-coding RNAs. PLoS Comput Biol.

[26] Schwemmle S, Pfeifer GP (2000). Genomic structure and mutation screening of the E2F4 gene in human tumors. Int J Cancer.

[27] Lee BK, Bhinge AA, Iyer VR (2011). Wide-ranging functions of E2F4 in transcriptional activation and repression revealed by genome-wide analysis. Nucleic Acids Res.

[28] Souza RF, Yin J, Smolinski KN, Zou TT, Wang S, Shi YQ, Rhyu MG, Cottrell J, Abraham JM, Biden K, Simms L, Leggett B, Bova GS, Frank T, Powell SM, Sugimura H, Young J, Harpaz N, Shimizu K, Matsubara N, Meltzer SJ (1997). Frequent mutation of the E2F-4 cell cycle gene in primary human gastrointestinal tumors. Cancer Res.

[29] Whitfield ML, Sherlock G, Saldanha AJ, Murray JI, Ball CA, Alexander KE, Matese JC, Perou CM, Hurt MM, Brown PO, Botstein D (2002). Identification of genes periodically expressed in the human cell cycle and their expression in tumors. Mol Biol Cell.

[30] Barrett T, Edgar R: Gene expression omnibus: microarray data storage, submission, retrieval, and analysis. *Methods Enzymol* 2006, 411:352–369. [http://www.ncbi.nlm.nih.gov/geo/], 10.1016/S0076-6879(06)11019-8 PMC1619900 16939800

[31] Ur-Rehman S, Gao Q, Mitsopoulos C, Zvelebil M: ROCK: a resource for integrative breast cancer data analysis. *Breast Cancer Res Treat* 2013, 139:907–921., 10.1007/s10549-013-2593-z 23756628

[32] Pubmed. [www.pubmed.com]

[33] Loi S, Haibe-Kains B, Desmedt C, Wirapati P, Lallemand F, Tutt AM, Gillet C, Ellis P, Ryder K, Reid JF, Daidone MG, Pierotti MA, Berns EM, Jansen MP, Foekens JA, Delorenzi M, Bontempi G, Piccart MJ, Sotiriou C (2008). Predicting prognosis using molecular profiling in estrogen receptor-positive breast cancer treated with tamoxifen. BMC Genomics.

[34] Desmedt C, Piette F, Loi S, Wang Y, Lallemand F, Haibe-Kains B, Viale G, Delorenzi M, Zhang Y, d’Assignies MS, Bergh J, Lidereau R, Ellis P, Harris AL, Klijn JG, Foekens JA, Cardoso F, Piccart MJ, Buyse M, Sotiriou C, Consortium T (2007). Strong time dependence of the 76-gene prognostic signature for node-negative breast cancer patients in the TRANSBIG multicenter independent validation series. Clin Cancer Res.

[35] Schmidt M, Bohm D, von Torne C, Steiner E, Puhl A, Pilch H, Lehr HA, Hengstler JG, Kolbl H, Gehrmann M (2008). The humoral immune system has a key prognostic impact in node-negative breast cancer. Cancer Res.

[36] Kim WJ, Kim EJ, Kim SK, Kim YJ, Ha YS, Jeong P, Kim MJ, Yun SJ, Lee KM, Moon SK, Lee SC, Cha EJ, Bae SC (2010). Predictive value of progression-related gene classifier in primary non-muscle invasive bladder cancer. Mol Cancer.

[37] Lee Y, Scheck AC, Cloughesy TF, Lai A, Dong J, Farooqi HK, Liau LM, Horvath S, Mischel PS, Nelson SF (2008). Gene expression analysis of glioblastomas identifies the major molecular basis for the prognostic benefit of younger age. BMC Med Genomics.

[38] Lee ES, Son DS, Kim SH, Lee J, Jo J, Han J, Kim H, Lee HJ, Choi HY, Jung Y, Park M, Lim YS, Kim K, Shim Y, Kim BC, Lee K, Huh N, Ko C, Park K, Lee JW, Choi YS, Kim J (2008). Prediction of recurrence-free survival in postoperative non-small cell lung cancer patients by using an integrated model of clinical information and gene expression. Clin Cancer Res.

[39] Smith JJ, Deane NG, Wu F, Merchant NB, Zhang B, Jiang A, Lu P, Johnson JC, Schmidt C, Bailey CE, Eschrich S, Kis C, Levy S, Washington MK, Heslin MJ, Coffey RJ, Yeatman TJ, Shyr Y, Beauchamp RD (2010). Experimentally derived metastasis gene expression profile predicts recurrence and death in patients with colon cancer. Gastroenterology.

[40] Bullinger L, Dohner K, Bair E, Frohling S, Schlenk RF, Tibshirani R, Dohner H, Pollack JR (2004). Use of gene-expression profiling to identify prognostic subclasses in adult acute myeloid leukemia. N Engl J Med.

[41] Hummel M, Bentink S, Berger H, Klapper W, Wessendorf S, Barth TF, Bernd HW, Cogliatti SB, Dierlamm J, Feller AC, Hansmann ML, Haralambieva E, Harder L, Hasenclever D, Kuhn M, Lenze D, Lichter P, Martin-Subero JI, Moller P, Muller-Hermelink HK, Ott G, Parwaresch RM, Pott C, Rosenwald A, Rosolowski M, Schwaenen C, Sturzenhofecker B, Szczepanowski M, Trautmann H, Wacker HH (2006). A biologic definition of Burkitt’s lymphoma from transcriptional and genomic profiling. N Engl J Med.

[42] Galea MH, Blamey RW, Elston CE, Ellis IO (1992). The Nottingham Prognostic Index in primary breast cancer. Breast Cancer Res Treat.

[43] Adjuvant!Online. [http://www.adjuvantonline.com/index.jsp]

[44] Gerstein MB, Kundaje A, Hariharan M, Landt SG, Yan KK, Cheng C, Mu XJ, Khurana E, Rozowsky J, Alexander R, Min R, Alves P, Abyzov A, Addleman N, Bhardwaj N, Boyle AP, Cayting P, Charos A, Chen DZ, Cheng Y, Clarke D, Eastman C, Euskirchen G, Frietze S, Fu Y, Gertz J, Grubert F, Harmanci A, Jain P, Kasowski M (2012). Architecture of the human regulatory network derived from ENCODE data. Nature.

[45] Cheng C, Min R, Gerstein M (2011). TIP: a probabilistic method for identifying transcription factor target genes from ChIP-seq binding profiles. Bioinformatics.

[46] Sorlie T, Perou CM, Tibshirani R, Aas T, Geisler S, Johnsen H, Hastie T, Eisen MB, van de Rijn M, Jeffrey SS, Thorsen T, Quist H, Matese JC, Brown PO, Botstein D, Lonning PE, Borresen-Dale AL (2001). Gene expression patterns of breast carcinomas distinguish tumor subclasses with clinical implications. Proc Natl Acad Sci U S A.

[47] Parker JS, Mullins M, Cheang MC, Leung S, Voduc D, Vickery T, Davies S, Fauron C, He X, Hu Z, Quackenbush JF, Stijleman IJ, Palazzo J, Marron JS, Nobel AB, Mardis E, Nielsen TO, Ellis MJ, Perou CM, Bernard P (2009). Supervised risk predictor of breast cancer based on intrinsic subtypes. J Clin Oncol.

[48] Sorlie T, Borgan E, Myhre S, Vollan HK, Russnes H, Zhao X, Nilsen G, Lingjaerde OC, Borresen-Dale AL, Rodland E (2010). The importance of gene-centring microarray data. Lancet Oncol.

[49] Paik S, Shak S, Tang G, Kim C, Baker J, Cronin M, Baehner FL, Walker MG, Watson D, Park T, Hiller W, Fisher ER, Wickerham DL, Bryant J, Wolmark N (2004). A multigene assay to predict recurrence of tamoxifen-treated, node-negative breast cancer. N Engl J Med.

[50] DAVID (the Database for Annotation, Visualization and Integrated Discovery). [http://david.abcc.ncifcrf.gov/gene2gene.jsp]

[51] Rouzier R, Perou CM, Symmans WF, Ibrahim N, Cristofanilli M, Anderson K, Hess KR, Stec J, Ayers M, Wagner P, Morandi P, Fan C, Rabiul I, Ross JS, Hortobagyi GN, Pusztai L (2005). Breast cancer molecular subtypes respond differently to preoperative chemotherapy. Clin Cancer Res.

[52] Michiels S, Koscielny S, Hill C (2005). Prediction of cancer outcome with microarrays: a multiple random validation strategy. Lancet.

[53] Jenssen TK, Hovig E (2005). Gene-expression profiling in breast cancer. Lancet.

[54] Carroll JS, Prall OW, Musgrove EA, Sutherland RL (2000). A pure estrogen antagonist inhibits cyclin E-Cdk2 activity in MCF-7 breast cancer cells and induces accumulation of p130-E2F4 complexes characteristic of quiescence. J Biol Chem.

[55] Dhillon NK, Mudryj M (2002). Ectopic expression of cyclin E in estrogen responsive cells abrogates antiestrogen mediated growth arrest. Oncogene.

[56] Scholzen T, Gerdes J (2000). The Ki-67 protein: from the known and the unknown. J Cell Physiol.

[57] Ferguson NL, Bell J, Heidel R, Lee S, Vanmeter S, Duncan L, Munsey B, Panella T, Orucevic A (2013). Prognostic value of breast cancer subtypes, Ki-67 proliferation index, age, and pathologic tumor characteristics on breast cancer survival in Caucasian women. Breast J.

[58] Nagasako Y, Misawa K, Kohashi S, Hasegawa K, Okawa Y, Sano H, Takada A, Sato H (2003). Evaluation of malignancy using Ki-67 labeling index for gastric stromal tumor. Gastric Cancer.

[59] Hitchcock CL (1991). Ki-67 staining as a means to simplify analysis of tumor cell proliferation. Am J Clin Pathol.

