# Supplementary figures and images for: E2F4 regulatory program predicts patient survival prognosis in breast cancer
Source: Breast Cancer Res. 2014 Dec 2;16:486. doi: 10.1186/s13058-014-0486-7 (PMC4303196; doi:10.1186/s13058-014-0486-7)

**E2F4 activity, all subtypes  
n=1902**

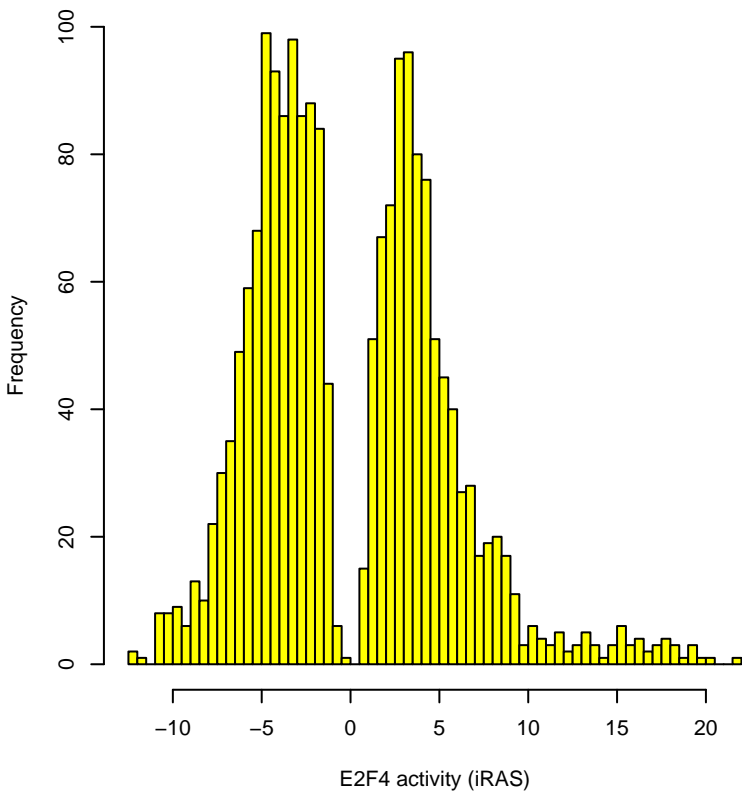

**E2F4 activity, ER Positive only  
n=1485**

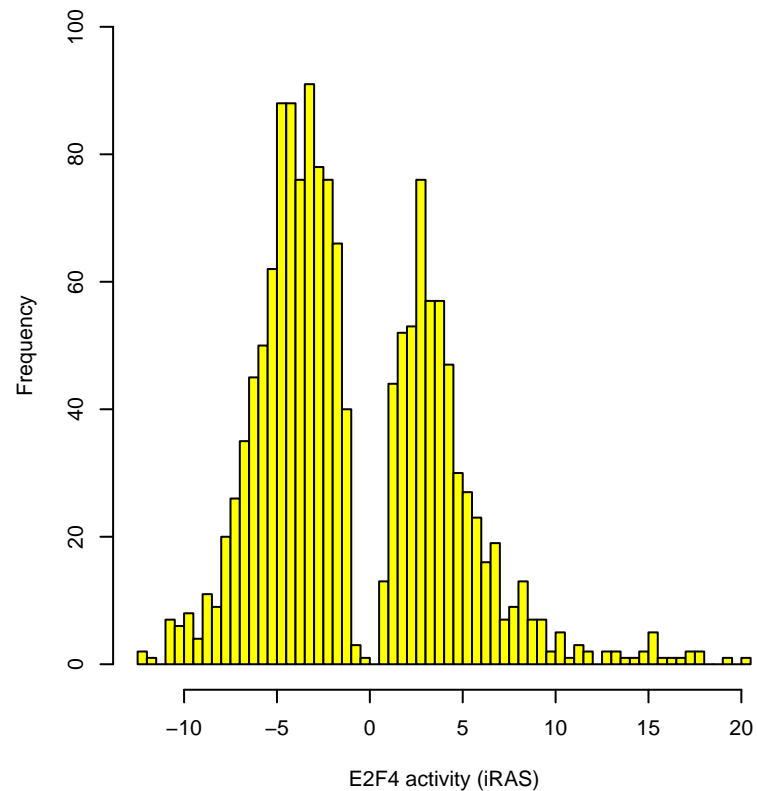

**E2F4 activity, ER Negative only  
n=393**

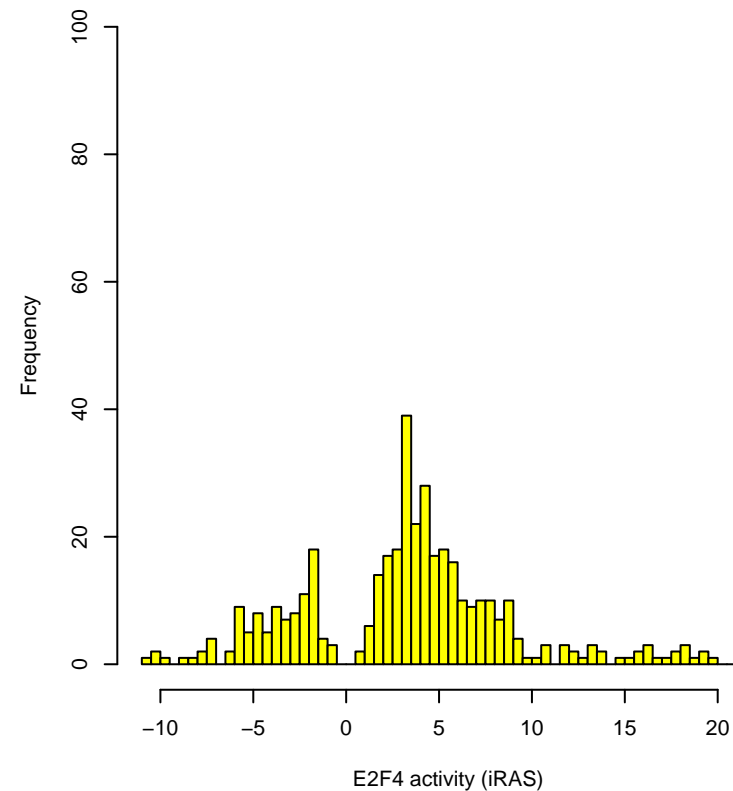

Supplement: Supplementary file 3 — Additional file 3: Figure S1.: The distribution of E2F4 scores in all (the left panel), ER+ (the middle panel) and ER- (the right panel) breast cancer samples. (PDF 6 KB) [file 13058_2014_486_MOESM3_ESM.pdf]

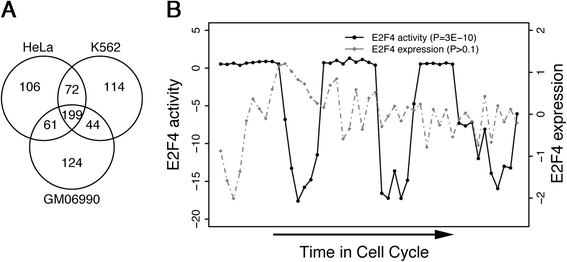

Supplement: Supplementary file 5 — Authors’ original file for figure 1 [file 13058_2014_486_MOESM5_ESM.gif]

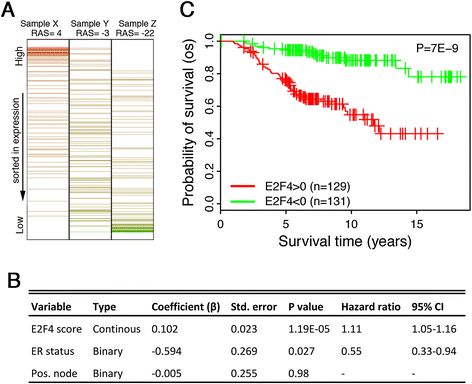

Supplement: Supplementary file 6 — Authors’ original file for figure 2 [file 13058_2014_486_MOESM6_ESM.gif]

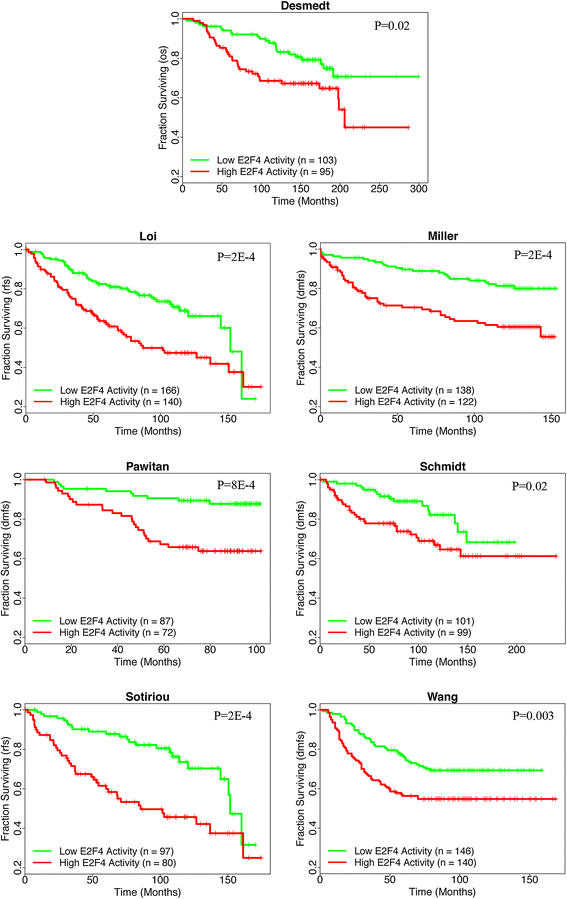

Supplement: Supplementary file 7 — Authors’ original file for figure 3 [file 13058_2014_486_MOESM7_ESM.gif]

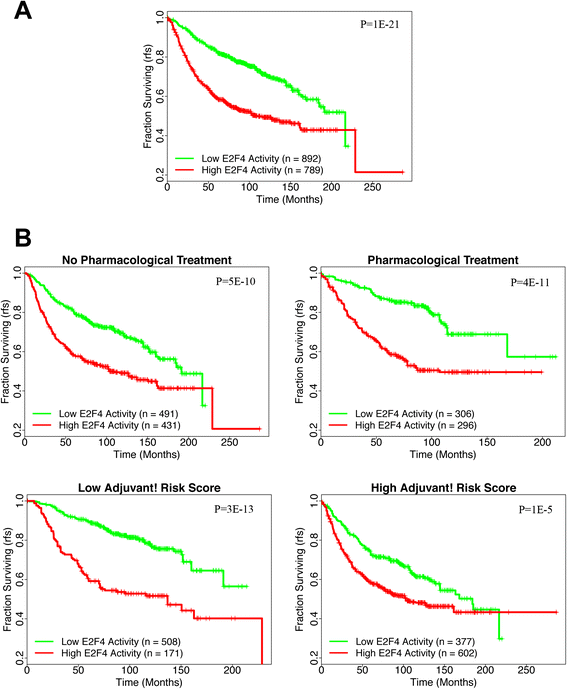

Supplement: Supplementary file 8 — Authors’ original file for figure 4 [file 13058_2014_486_MOESM8_ESM.gif]

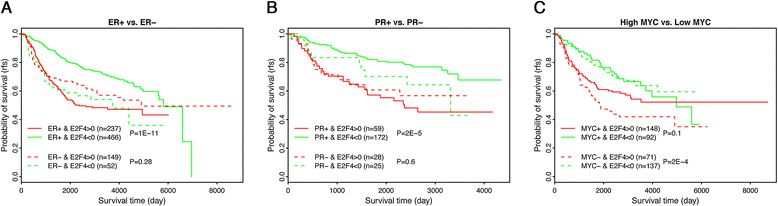

Supplement: Supplementary file 9 — Authors’ original file for figure 5 [file 13058_2014_486_MOESM9_ESM.gif]

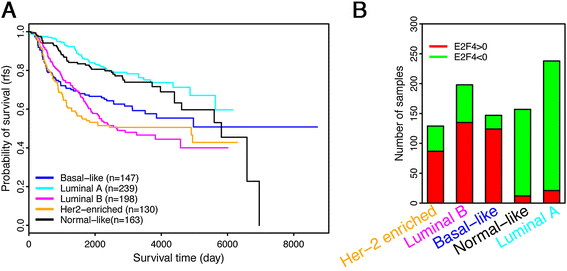

Supplement: Supplementary file 10 — Authors’ original file for figure 6 [file 13058_2014_486_MOESM10_ESM.gif]

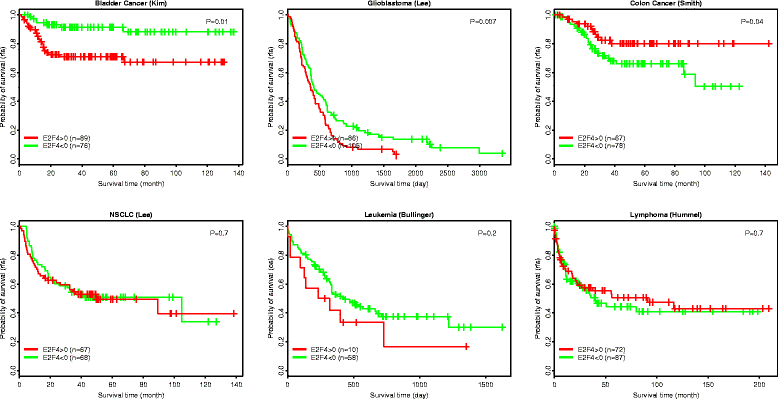

Supplement: Supplementary file 11 — Authors’ original file for figure 7 [file 13058_2014_486_MOESM11_ESM.gif]
