# Supplementary material for: E2F4 regulatory program predicts patient survival prognosis in breast cancer
Source: Breast Cancer Res. 2014 Dec 2;16:486. doi: 10.1186/s13058-014-0486-7 (PMC4303196; doi:10.1186/s13058-014-0486-7)
Supplement: Supplementary file 4 — Additional file 4: Figure S2.: Application of E2F4 signature to predicting prognosis of ER+ node-breast cancer. Top left: Oncotype DX divides samples into high-, intermediate- and low-relapse risk groups. Top right: E2F4 signature divides patient into two groups with significant survival difference. Bottom left: E2F4 can further stratify the Oncotype-classified intermediate group into high- and low-risk groups. Bottom right: Within the Oncotype-classified intermediate group, patients with high and low Oncotype DX scores do not show significant difference in their survival times. (PDF 21 KB) [file 13058_2014_486_MOESM4_ESM.pdf]

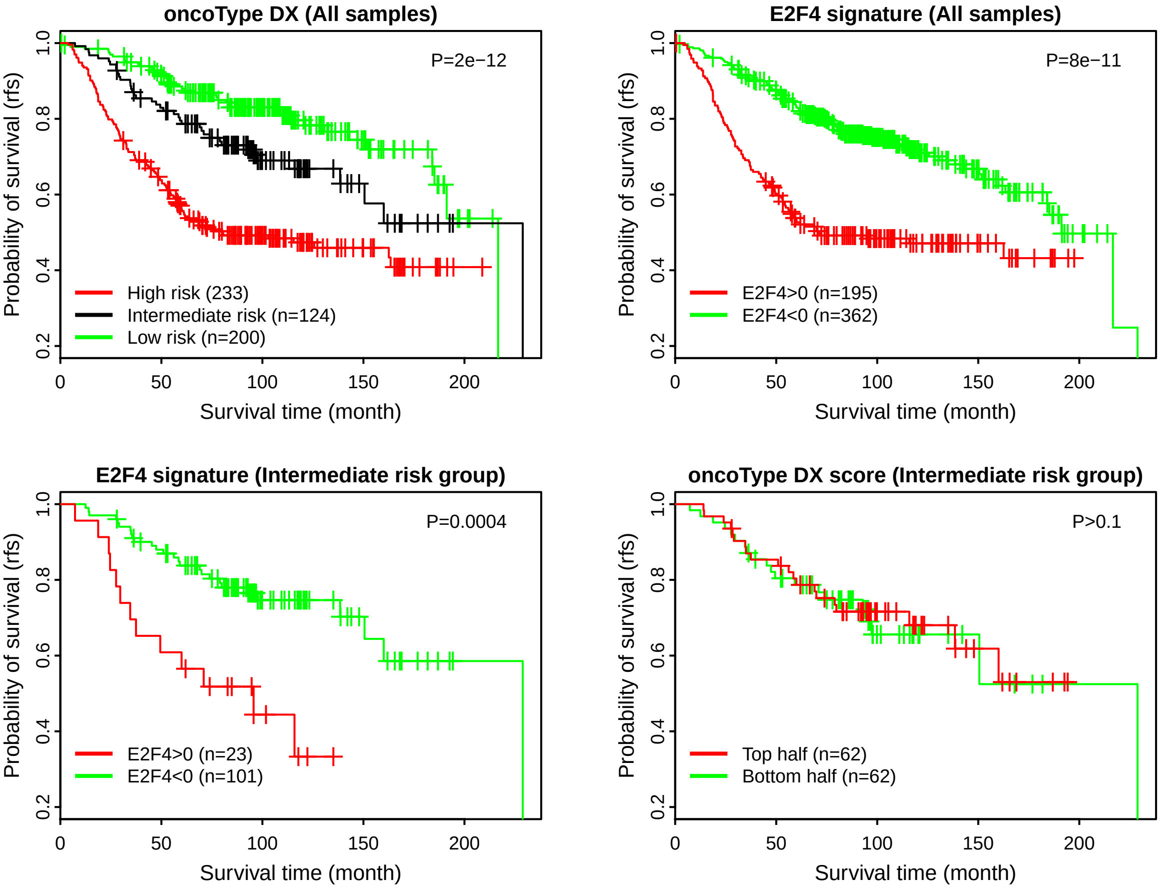

oncoType DX (All samples)
P=2e−12
High risk (233)
Intermediate risk (n=124)
Low risk (n=200)
Probability of survival (rfs)
Survival time (month)
0.2
0.4
0.6
0.8
1.0
0
50
100
150
200
E2F4 signature (All samples)
P=8e−11
E2F4>0 (n=195)
E2F4<0 (n=362)
E2F4 signature (Intermediate risk group)
P=0.0004
E2F4>0 (n=23)
E2F4<0 (n=101)
oncoType DX score (Intermediate risk group)
P>0.1
Top half (n=62)
Bottom half (n=62)
